# “We don’t want problems”: reasons for denial of legal abortion based on conscientious objection in Mexico and Bolivia

**DOI:** 10.1186/s12978-021-01101-2

**Published:** 2021-02-17

**Authors:** Stephanie Andrea Küng, Jasmine Danette Wilkins, Fernanda Díaz de León, Freddy Huaraz, Erin Pearson

**Affiliations:** 1 Ipas, Chapel Hill, NC USA; 2 Ipas Central America and Mexico, Mexico City, Mexico; 3 Ipas Bolivia, La Paz, Bolivia

**Keywords:** Abortion, Latin America and the caribbean, Conscientious objection, Public health, Law and policy

## Abstract

**Background:**

The misuse of conscientious objection (CO) is a significant barrier to legal abortion access in many countries, especially in Latin America. We examine the reasons for denial of legal abortion services in Mexico and Bolivia and identify ways to mitigate the misuse of CO.

**Methods:**

We conducted 34 in-depth interviews and 12 focus group discussions in two states in Mexico and four departments in Bolivia. Results were coded and categorized using a thematic analysis approach.

**Results:**

Denial of abortion services based on CO is widespread in health facilities in Mexico and Bolivia and is primarily employed for reasons other than moral, religious, or ethical considerations. The main reasons for denial of services based on CO is lack of knowledge about abortion-related laws and fear of legal problems in abortion service provision. Conversely, the main reason *to* provide services is to comply with relevant laws. Denying services under the guise of CO negatively impacts pregnant people and health care teams, including fewer safe abortion options and increased workload and stigma, respectively. Most respondents cited training and education on abortion law as the foremost way to mitigate the negative impacts of the misuse of CO.

**Conclusions:**

For many health personnel, knowing, understanding, and following the law is reason enough to provide abortion services. Individuals who object due to lack of knowledge about laws and fear of legal problems represent a key population that can be sensitized and equipped with the necessary information and resources to provide legal abortion services.

## Resumen

**Antecedentes:**

El mal uso de la objeción de conciencia (OC) es una barrera importante para el acceso al aborto aún cuando es legal, en muchos países, especialmente en países en América Latina. Examinamos los motivos de la negación de servicios de aborto legal en México y Bolivia e identificamos formas de mitigar el uso indebido de la OC.

**Métodos:**

Realizamos 34 entrevistas a profundidad y 12 discusiones en grupo focal en dos estados en México y cuatro departamentos en Bolivia. Los resultados fueron codificados y categorizados utilizando un enfoque de análisis temático.

**Resultados:**

La negación de servicios de aborto basados ​​en la OC está muy extendida en los establecimientos de salud en México y Bolivia y se emplea principalmente por razones distintas a las consideraciones morales, religiosas o éticas. Las principales razones para la negación de servicios basados ​​en la OC son la falta de conocimiento sobre las leyes relacionadas con el aborto y el temor a problemas legales en la prestación de servicios de aborto. Por el contrario, la razón principal para proporcionar servicios es cumplir con las leyes pertinentes. Negar servicios bajo la apariencia de OC impacta negativamente a las personas embarazadas y a los equipos de atención médica, incluidas menos opciones de aborto seguro y mayor carga de trabajo y estigma, respectivamente. La mayoría de los encuestados mencionaron la capacitación y educación sobre la ley del aborto como la principal forma de mitigar los impactos negativos del uso indebido de la OC.

**Conclusiones:**

Para parte del personal de salud, conocer, comprender y cumplir la ley es motivo suficiente para proporcionar servicios de aborto. Las personas que se oponen debido a la falta de conocimiento sobre las leyes y el miedo a los problemas legales representan una población clave que puede ser sensibilizada y equipada con la información y los recursos necesarios para proporcionar servicios de aborto legal.

## Plain English summary

In Bolivia and the Mexican States of Jalisco and Mexico State, abortion is legal under certain indications. However, health care providers have the legal right to claim conscientious objection (CO) when a service conflicts with their moral, religious, or ethical beliefs. Evidence shows that CO is often invoked to refuse to participate in legal abortion services, but it also shows that this objection is often *not* based in moral, religious, or ethical reasons. Our study seeks to identify reasons for denial of abortion services in Mexico and Bolivia, where information about the reasons for invoking CO is lacking. Our study benefits from the participation of health care personnel who self-identify as objectors.

We conducted 34 in-depth interviews (IDIs) and 12 focus group discussions (FGDs) in Mexico and Bolivia to explore the understanding and use of CO and ways to mitigate the negative impacts of CO on access to legal abortion services.

We found that the main reasons for denial of services based on CO among our sample is lack of knowledge about abortion-related laws and fear of legal problems in abortion service provision. Conversely, the main reason *to* provide services is to comply with relevant laws. Efforts to expand access to legal abortion in these settings must understand and respond to these principal motivators in order to decrease misuse of CO.

## Background

The misuse of conscientious objection (CO) in denying abortion services has emerged as a significant barrier to accessing legal abortion in many countries, especially in Latin America, which has some of the most restrictive abortion laws in the world [1, 2]. CO is the right of an individual to refuse to participate in any activity deemed incompatible with their deeply held moral, religious, or ethical beliefs [3]. Evidence suggests that CO is sometimes improperly used as a justification by public sector health care providers and institutions alike to exempt themselves from their professional responsibility to provide essential reproductive health services, including abortion care, as required by international and national laws, policies, and protocols [4–6].

Studies have shown that in some settings providers invoke CO to refuse to provide legal abortion services, when in reality, their objection is based on misunderstanding and misinformation, lack of trust in the person requesting abortion services, fear of police harassment or legal punishment, peer pressure or broader social stigma, and/or economic gain. Such a range of rationales makes CO a complex issue [4, 6–8].

The data presented here are from a larger effort conducted by Ipas to understand how abortion-related CO manifests in Bolivia, Mexico, and South Africa and ultimately, to create and test interventions that mitigate the negative effects of CO. Ipas is an international non-governmental organization that works in affiliation with public hospitals around the world to prevent unsafe abortion through technical assistance and training. Despite the breadth of information about the use of CO in diverse settings, information was lacking from the regions where we planned to implement our tailored interventions. Due to differences in methodologies between the three countries, results from South Africa will be published separately.

Invoking CO as grounds to refuse to provide legal abortion services violates the right to health as established in international human rights norms. United Nations (UN) treaty monitoring bodies, the Inter-American Commission on Human Rights (IACHR), and the International Federation of Gynecology and Obstetrics (FIGO) Committee for the Ethical Aspects of Human Reproduction and Women's Health all recognize the primacy of health care access over provider objection, the need for effective health systems that guarantee legal reproductive health care access, and the responsibility of providers, facilities, and governments to ensure and uphold such access [9–11]. In both Mexico and Bolivia, abortion is only legal under certain indications, except in Mexico City and the Mexican state of Oaxaca, where abortion upon request is legal during the first trimester [12]. Federal law in Mexico allows health care providers (doctors and nurses), to invoke CO except when there is a risk to the person’s life or when it is a medical emergency [13]. Additionally, the federal-level policy known as Official Mexican Standard 046 on Domestic and Sexual Violence and Violence against Women (NOM 046) established that public institutions must have non-objecting providers available at all shifts to provide abortion services [13, 14]. In Bolivia, the right to and limitations on the exercise of abortion-related CO are included in national guidelines as an individual (not institutional) right. These same guidelines establish that legal abortion services must be provided within 24 h following refusal by an objecting provider [15].

Our objective was to examine the understanding, use, and impacts of CO among public sector physicians and other allied health personnel in select regions in Mexico and Bolivia, and to use this information to create and test innovative interventions to mitigate the negative impacts of CO on access to legal abortion services. Post-abortion care (PAC) services are considered emergency services and are largely provided in Mexico and Bolivia without objection. The data presented here are from the first phase of this effort, which included formative research conducted through in-depth interviews and focus group discussions with health personnel in Mexico and Bolivia. To our knowledge, no published literature exists studying the exercise of CO among physicians and other allied health personnel identifying as both non-objectors *and* objectors in Mexican states outside of Mexico City and in Bolivia as a whole.

## Methods

Between December 2018 and June 2019, we conducted 34 in-depth interviews (IDIs) and 12 focus group discussions (FGDs) in Mexico and Bolivia to explore the understanding and use of conscientious objection (CO) and ways to mitigate the negative impacts of CO on access to legal abortion services. IDIs were conducted with 17 objecting and 17 non-objecting obstetricians/gynecologists and generalists (Table 1). 16 of the 34 IDIs (47%) were with physicians in Bolivia, while 18 (53%) were with physicians in Mexico. The majority (56%) of IDI respondents were women. Both in Mexico and Bolivia, most of the objectors were women (75% and 56%, respectively). FGDs were conducted with mixed groups of physicians and other allied health personnel (including hospital unit leads, nurses, legal, data and administrative personnel, and social workers); we conducted 4 in Bolivia and 8 in Mexico with an average of 7 participants per FGD and a total of 82 participants. One-third of FGD participants were nurses (including head nurses), one-quarter were doctors (27%), and the rest were legal, data and administrative personnel (23%) and psychologists and social workers (17%). The majority of FGD participants (76%) were women. All participants were employed in public-sector hospitals.

**Table 1.** Characteristics of the sample

	FGDs			IDIs		
	Mexico n = 50	Bolivia n = 32	Combined n = 82	Mexico n = 18	Bolivia n = 16	Combined n = 34
	n (%)	n (%)	n (%)	n (%)	n (%)	n (%)
Profession						
Nurse (including head nurses)	18 (36%)	9 (28%)	27 (33%)			
Administrative/data/legal personnel	7 (14%)	12 (38%)	19 (23%)			
Psychologist/social worker	3 (6%)	11 (34%)	14 (17%)			
Physician (gynecologist/obstetricians and generalists)	22 (44%)	0	22 (27%)	18 (100%)	16 (100%)	34 (100%)
Objectors				8 (44%)	9 (56%)	17 (50%)
Non-objectors				10 (56%)	7 (44%)	17 (50%)
Gender						
Male	14 (28%)	6 (19%)	20 (24%)	6 (33%)	9 (56%)	15 (44%)
Female	36 (72%)	26 (81%)	62 (76%)	12 (67%)	7 (44%)	19 (56%)

We chose to conduct IDIs and FGDs to capture both personal experiences and group norms of objecting and non-objecting doctors and allied health personnel. We conducted IDIs with doctors to understand better their personal experience with CO. We conducted FGDs with allied health personnel and physicians to get a sense of the hospital environment and foster a discussion of how they see CO invoked in their hospitals. IDIs and FGDs were conducted until data saturation was achieved. IDIs and FGDs were conducted by trained interviewers in Spanish. Interviewers were not affiliated with the hospitals and were not known to participants. The interviewer in Bolivia was Ipas staff, and interviewers in Mexico were external consultants; interviewer affiliation with Ipas was known to participants.

We recruited participants via convenience sampling; professional contacts in selected hospitals identified objecting and non-objecting providers to participate in the IDIs for this study. These contacts are members of health care teams and know who objecting providers are. In the case of FGDs, these same contacts identified additional physicians and allied health personnel (not categorized as objectors or non-objectors) to participate. All participants were then contacted in person to assess interest and obtain informed consent.

In Bolivia, we conducted IDIs and FGDs in four public hospitals located in four departments: Chuquisaca, La Paz, Potosí, and Santa Cruz. Abortion is legal in Bolivia in the case of rape, incest, risk to health, and fetal malformation [16]. In Mexico, we conducted IDIs and FGDs in four public hospitals located in two states: Jalisco and Mexico State. In the state of Jalisco, abortion is legal in the case of rape, accidental abortion, and risk to health or life. In Mexico State, abortion is legal in the case of rape, accidental abortion, risk to life, and congenital defects [12]. This study was the formative phase of a project aimed to develop interventions to address CO. As such, hospitals in both countries were chosen based on (1) their location in populous states/departments and, in the case of Mexico, states that represent a range of legal frameworks on abortion, making them strategic and priority regions for abortion-related CO interventions; (2) anecdotal yet verifiable reports of CO from hospital staff; and (3) institutional willingness and Ipas affiliation, which allowed for partnership in developing and testing CO-related interventions based on formative research findings.

One member of the research team with expertise in qualitative research methodology and familiarity with the subject matter developed Spanish-language semi-structured interview and FGD guides. These guides were edited in consultation with other members of the research team and pilot tested. We developed one FGD guide and separate IDI guides for objectors and non-objectors to more closely examine their nuanced understanding and use of CO. The guides focused on reasons for objecting to legal abortion services, barriers to legal abortion services, knowledge of CO guidelines and protocols, impacts of CO on patients and health care providers, and suggestions for mitigating the negative impacts of CO on legal abortion service provision. Objectors were asked more specifically about their objection, whereas non-objectors were asked to reflect on objection by their coworkers. Probes were used to further explore topics that emerged during interviews. As evidence shows that CO is mainly a problem for legal (induced) abortion services offered through the public health system [5, 17], interview guides focused on legal abortion and all participants worked at public hospitals. Interviewers fluent in Spanish conducted, recorded, and transcribed all interviews verbatim. Interviews were stored on password-protected devices and unique IDs were created to protect identifying information. Members of the research team listened to recordings periodically throughout data collection and reviewed transcriptions for quality.

Research team members fluent in Spanish analyzed transcriptions using a thematic analysis approach. The first author read all transcripts and developed an initial codebook using themes that emerged from the data. This codebook was then reviewed and edited in consultation with three other members of the research team. Descriptive memos were used to identify other themes and add them to the codebook following consensus among research team members. After finalizing the codebook, two researchers double-coded transcripts to check for inter-coder reliability and made final edits and clarifications to the codebook as needed. Coding and analysis were done using ATLAS.ti version 7.5.12. Multilingual members of the research team translated all quotes from Spanish into English applying meaningful translation rather than literal translation to more appropriately convey message or meaning. This study received institutional review board (IRB) approval from the Allendale Investigational Review Board (AIRB).

## Results

Our analysis reveals that, according to respondents, conscientious objection (CO) is widespread across public hospitals providing legal (induced) abortion services in Mexico and Bolivia; however, there is a lack of consensus regarding the pervasiveness of denial of abortion services more broadly. When providers were asked to estimate the percentage of physicians in their hospitals that would refuse to provide legal abortion services in certain situations, answers ranged from 10% to 95%. This wide range hints at variation across hospitals and regions, lack of transparency around objector status within hospitals, and nuances of refusing services in certain situations as opposed to all. Key here is that *all* interviewees knew of providers who would refuse to provide legal abortion in certain situations. Importantly, misuse of CO was identified as an issue mainly in public hospitals. This was largely attributed to differences in economic incentives between providers delivering services in public facilities compared with private practices. Providers do not receive payment for abortion services delivered in public hospitals as salaried employees, while providers receive most, if not all, payment for services rendered in their private practices. Many non-objecting providers used the term “double standard” to refer to providers who work in both the public and private settings but are only willing to provide abortion services in their private practice. As one provider in Bolivia stated,

*I see this as, as being two-faced, it’s about showing publicly that they don’t want to do something, when perhaps, they do it privately – a double standard. [Non-objector, Chuquisaca, Bolivia]*

### Reasons for objecting

Across the board, physicians and other allied health personnel interviewed in Mexico and Bolivia correctly defined CO in terms of moral, religious, or ethical objections to providing abortion services. However, when interviewees were asked about specific reasons why they or other providers object to providing legal abortion services, we found a wide variety of reasons and interpretations that fall outside the scope of CO as legally defined. The following sections highlight the most common reasons that emerged for denying legal abortion services across both countries.

#### Lack of knowledge about laws and protocols

Despite correctly defining CO, there is widespread lack of knowledge about laws, policies, protocols, and procedures that clarify and standardize the exercise of CO. Almost no respondents correctly identified whether and how the practice of CO is regulated in their countries. There is a similar lack of knowledge about laws that define when abortion can be legally provided. While some providers in Mexico and Bolivia correctly identified the legal indications for abortion in their respective contexts, confusion and uncertainty about abortion-related laws and policies remains widespread. Many interviewees either incorrectly defined abortion-related laws and policies or shared that many of their colleagues are unclear about them. In particular, there was confusion about required documentation for accessing abortion care in the case of rape in Mexico and Bolivia, with many respondents incorrectly describing the type of documentation required. In Bolivia, where rape is a legal indication for abortion, an earlier iteration of the law required rape survivors to file an official incident report with the police and have this report signed by a judge before being allowed to access abortion care in a public health facility. This requirement was modified in 2014. Now, the official incident report required to access abortion care at a public facility in the case of rape *no longer* has to be signed by a judge [18]. In Mexico, NOM 046 holds that rape survivors do not need to file an official incident report with legal authorities in order to access abortion care for any pregnancy resulting from rape; they need only indicate that they have experienced rape when seeking abortion care at any public health facility [14]. However, this federal policy often conflicts with state-level laws and policies (although not in the two states in this study), and there is persistent lack of knowledge and understanding about the policy. This quote from a provider in Mexico exemplifies this confusion,

*…before the regulatory change, for a patient to be able to access legal abortion, they had to provide supporting documentation from the public prosecutor’s office, that is, a filed complaint or proof of filing with the public prosecutor’s office demonstrating that the prosecutor’s office knew that this patient had been sexually abused and for that reason became pregnant…but this change [in the federal policy] has been very difficult for doctors to understand. *[Objector, Mexico State, Mexico]

#### Fear of legal problems and lack of legal support

Directly linked to lack of knowledge and understanding of laws, policies, protocols, and procedures is widespread fear of “getting involved” or “getting mixed up” in abortion service provision. Providers end up avoiding abortion services altogether due to uncertainty about abortion-related laws and policies and their responsibilities under them, as well as contradictions between laws and policies enacted at different levels of government (federal, state, local), especially in the case of Mexico. As one provider in Mexico stated,

*The problem is that they don’t want legal issues. Because the penal code does not align with the Official Mexican Standard [NOM 046], there is tension surrounding whether to provide [abortion care]. We don’t want legal problems. That’s the issue. It’s not that we are so religious, far from it.* [Non-objector, Jalisco, Mexico]

The same sentiment was shared among providers in Bolivia. As one provider shared,

*There are colleagues who say it’s because of conscientious objection that they don’t do it [provide abortion care], but let’s just say that this is an out…they don’t want to do it because they don’t want to complicate things. It’s not really because of their religion or something, it’s just to avoid problems in the long run.* [Non-objector, Santa Cruz, Bolivia]

Specifically, providers in these countries fear medical malpractice claims, complaints, and lawsuits resulting from their provision of abortion services, as demonstrated by these quotes,

*There is this vulnerability around, what are the rules or norms or steps that a patient legally has to take so that one can provide care [an abortion], because later something happens, some complication with the patient and then, how will the doctor vindicate themselves? The patient or their family could sue…to protect ourselves we want to know what is legal, under what law are we going to protect ourselves, right? If something happens to the patient, how will the doctor shield themselves?* [Non-objector, Santa Cruz, Bolivia]

*Patients have a lot of power to lodge a complaint, to complain about the abortion care they received. Unfortunately, some of my doctors have had a bad experience being subjected to a lawsuit or a complaint that requires you to justify your actions, and that puts them on alert.* [Objector, Mexico State, Mexico]

The fact that legal abortion services are rarely requested by patients in these public hospitals contributes to ongoing lack of familiarity and knowledge of its existence among providers. Nearly all interviewed providers spoke about the low demand for legal abortion services in their respective hospitals,

*The majority of patients who come here arrive having already induced abortion, and you can’t do anything else besides perform the procedure. That’s the reality in this hospital. The other cases [legal abortion] are minimal.* [Deputy Medical Director, La Paz, Bolivia]

#### Provider value judgements

In Bolivia, we found that providers felt most discomfort around providing abortion care in the case of rape. This discomfort seems to stem from providers being mistrustful of patients who cite rape as the legal indication under which they seek abortion care, especially since requirements for accessing legal abortion services in the case of rape have been relaxed in Bolivia. As one objecting provider stated,

*After the training I think they even wanted us to just, just do it [provide abortion services] upon a patient's request. I told them at the time that most patients will come and say, "I’ve been raped", even if they haven’t been raped…so, just imagine how many abortions they’re going to do each day.* [Objector, Potosí, Bolivia]

Providers in both Mexico and Bolivia also expressed discomfort with providing abortion care when they perceive that a patient acted irresponsibly and became pregnant as a result of not taking certain precautions. More pointedly, they blame the patient for not leveraging what they perceive to be readily available and accessible contraceptive services and methods.

*I mean, in the case of, I got pregnant because I didn’t take the pill, because I forgot, because I was drunk, I think that is where it doesn’t count, no? I think, I mean, regardless of whether the patient has the right to choose, there are also contraceptive methods…that is, if you are going to have an active sex life…and as part of your life plan you don’t want to get pregnant, well, why don’t you use a contraceptive method? *[Objector, Mexico State, Mexico]

*Am I for or against it? It depends on the reason, no? The reason why that pregnancy happened in the first place…Because, normally, all health clinics provide family planning, for free, so it’s necessary for me to know the reason for this pregnancy for me to see if, because even here in Bolivia that’s not permitted, abortion on demand, and I still do not support that.* [Objector, Santa Cruz, Bolivia]

#### Gestational age and fetal viability

Our research also revealed reasons for objecting that were tied to gestational age and fetal viability. As a provider in Mexico expressed,

*…but when they are patients with more advanced pregnancies… I know that I won’t be able to control my emotions…I say, “Well, I can’t help you…”* [Non-objector, Mexico State, Mexico]

For some objectors in Bolivia, this was closely tied to their belief that the fetus is a human life that should be valued and protected. This belief was much less prevalent in our interviews with providers and other allied health personnel in Mexico. Belief that the fetus is a life that must be protected was one of the only reasons uncovered in our analysis that fits under the legal definitions of CO, and was communicated in the following ways:

*Well, no one has the right to take the life of another human being, right?* [Objector, Santa Cruz, Bolivia]

*…there is a life that is present and totally innocent and is not at fault for being alive, no?* [Objector, Chuquisaca, Bolivia]

Respondents in Bolivia also linked the belief that the fetus is a life with a medical responsibility to protect that life, as expressed in the following quotes:

*The principle of the Hippocratic Oath has always prevailed, right? To defend life, from the moment of conception* [Objector, La Paz, Bolivia]

*I think that in some cases it’s the issue, the moral issue, the religious issue, because it [the fetus] doesn’t stop being a life, no? And, since our oath tells us to “save lives” I think it’s that.* [Objector, Santa Cruz, Bolivia]

### Reasons to provide abortion

#### Compliance with the law

Non-objecting providers in Mexico and Bolivia identified compliance with the law as the main reason for providing legal abortion services. As one non-objecting provider in Bolivia stated,

*The part of your professional duties for which you have been trained, to serve the patient without judging, criticizing, none of that, so, if a patient comes and it’s within the law, you have to do it…* [Non-objector, Potosí, Bolivia]

Interestingly, defense of a person’s right to an abortion or to make decisions about their own bodies more broadly was much less commonly cited by health care providers in both countries. When the right to a legal abortion was mentioned, it was often discussed in terms of consequences of being denied legal abortion care—for example, death or disability from unsafe abortion.

#### Medical responsibility

While some objectors in Mexico and most objectors in Bolivia cited their duty to protect the life of the fetus as justification for refusing to provide abortion services, non-objectors in both countries instead used medical responsibility to justify their *provision* of abortion services. However, non-objectors have a different understanding of *whose* well-being they as physicians are responsible for safeguarding, focusing on the pregnant person as opposed to the fetus:

*How many clandestine clinics are there all around us? How many patients come to us punctured, infected, with problems. Do you prefer that to them doing it like they should? Without problems, medically, without running the risk of the woman dying. Because that is one of the objectives, to lower the morbidity and mortality of pregnant women.* [Non-objector, Mexico State, Mexico]

### Profiles of CO users

Because abortions in Mexico and Bolivia are only performed by physicians^1^ [18, 19], all interviewees identified physicians as the primary decision-makers when it comes to abortion service provision and, therefore, the main obstacle to abortion access in the case of objection. However, it is important to note that objecting physicians are not the only ones who can be obstacles to receiving timely and high-quality legal abortion care in public facilities. Interviewees identified several other personnel, including nurses, social workers, front desk or reception staff, and police officers or security staff posted at hospital entrances. These individuals may be either unfamiliar or not up to date with the most recent laws and policies—including legal indications for abortion, required documentation, and/or insurance coverage—and thus unintentionally provide misinformation or turn away individuals seeking abortion care. However, most respondents acknowledged that some of these personnel, despite not being able to legally claim CO, appropriate CO and intentionally hinder abortion access. They may do this by illegally requiring an official incident report in the case of rape, telling patients that abortion care is not provided at a given facility, and/or otherwise mistreating patients seeking abortion care. As one provider working in Mexico recounted,

*Yeah, it was a 20-something-year-old girl who came and requested a legal abortion but, well, practically all my colleagues said that we don’t do that here, and they told her to go to Mexico City *[Non-objector, Mexico State, Mexico]

The following comment from a provider in Bolivia also exemplified the mistreatment to which some patients are subjected by hospital staff when receiving abortion care,

*The main problem is all the obstacles at first, obstacles in the sense that they blame the woman for the pregnancy and many times they make value judgements, saying that they are patients who get pregnant for the hell of it. They got pregnant, and now that they can’t benefit from the situation, they look to have an abortion.* [Non-objector, Chuquisaca, Bolivia]

Other individuals with decision-making power also knowingly and unknowingly influence the ways in which people can access legal abortion services. In Mexico and Bolivia, many facilities still require individuals seeking abortion care in the case of rape to file and provide signed proof of an official incident report even though documentation is either not required or requirements have been relaxed. As one provider in Mexico stated,

*Well, at least here in Mexico State it’s still the case that many physicians who work in facilities like this one say that, yes, there are individuals that are requesting, well, that they [patients] come with the document from the public prosecutor’s office, no? Because they say, ‘well, and what if it’s not [rape] and what if something happens?’ *[Objector, Mexico State, Mexico]

Additionally, decisions impacting whether someone can access abortion services may also come from further up the chain of command within individual facilities. If those making the decisions are objectors, their influence can severely limit access to these services. In Mexico, some interviewees discussed myriad administrative barriers to accessing abortion care, while in Bolivia, they mentioned how patient requests for abortion services are often referred to the hospital’s medical board and reviewed on a case-by-case basis prior to service delivery. In some cases, the hospital director may also have final decision-making authority over whether abortion care is provided at all.

*Here it’s an obstacle, no? Because the administration sees it [abortion] as bad, because of their personal, philosophical understanding. So the dismissal of the patient begins there. *[Non-objector, Mexico State, Mexico]

*The hospital still convenes the medical board prior to providing legal abortion services, in which they analyze the case, they discuss expectations, consider the realities of the situation, and then, among two, three, or four doctors decide how to proceed…* [Non-objector, Chuquisaca, Bolivia]

*No, in reality, this worries me a little because it [the decision] becomes the sole responsibility of one person, who is the Director of Clinical Services…. If he [the Director] says no, that’s that. *[Objector, Chuquisaca, Bolivia]

### Impacts of CO

Most interviewees acknowledged that misuse of CO impacts people seeking legal abortion care in Mexico and Bolivia. Across both countries the most frequently cited effects were psychological impacts, with many respondents underscoring the depression and frustration that they believe patients may experience after being turned away from legal abortion services. Many interviewees also acknowledged that these psychological impacts may lead to significantly worse outcomes for individuals who are denied legal abortion care, including death or injury from unsafe abortion and attempted or completed suicide, as illustrated in the following quotes:

*Well, I think that…when someone is denied [abortion care], I think that, yes, there are…feelings of fear, panic, guilt, loneliness, anguish, suicidal ideation, depressive episodes, it’s a decision that is being refused, right? And making them assume forced motherhood… *[Psychologist, Potosí, Bolivia]

*So it’s the family – or the person – that feels frustrated, deceived, resigned after it all, or, on the other hand, chooses to go to, let’s say, to where there are ladies who perform abortion, and not in sanitary conditions, but in, in other conditions, no? One time a patient came to me with a stick inserted into their uterus, do you understand me? For what? To get rid of the baby, look, we go to the other extreme.* [Social Worker, Potosí, Bolivia]

Furthermore, some interviewees mentioned that after being denied abortion services many individuals choose to self-induce abortion by procuring pills, such as misoprostol, at pharmacies or via formal and informal internet vendors. Respondents also noted the effects of CO on timely care. Many described the lengthy delays that patients are forced to experience when requesting abortion services when a non-objecting provider is either not present or not available. Oftentimes patients must wait in the hospital until the next shift or even the next day to finally receive time-sensitive services.

Some, but not all, interviewees also acknowledged how CO affects other providers, especially non-objecting providers. The most commonly cited effects on other providers were the stress of an increased workload and the stigma of being labeled as “the abortion doctor.” As shared by two providers,

*It [CO] saturates us, overwhelms the unit and there too the stress of the doctors that are overwhelmed also influences the quality of care, no?* [Non-objector, Santa Cruz, Bolivia]

*Well yes, they stereotype – your own colleagues, they label you as the baby-killer, negative things, you know. Baby-killer, pro-abortion, derogatory things…* [Non-objector, Mexico State, Mexico]

Interestingly, these effects were more readily acknowledged by non-objecting providers; most objecting providers did not identify or acknowledge the possible effects of their objection on other providers.

#### Mitigating the impact of CO

We also asked providers and other allied health personnel what is needed to mitigate the negative impacts of CO on people requesting legal abortion services. By and large, they expressed the need for sensitization and training on abortion as an essential healthcare service, abortion-related laws and policies, and the exercise and limits of CO.

*…ongoing training on these topics, as we’ve seen we have shortcomings, we don’t have knowledge of many regulations, that if we knew we could provide services, no? We don’t have much knowledge.* [Administrator, Potosí, Bolivia]

*…and we should be trained to help, right? Because sometimes we’re not even trained, no? Why? Because we don’t even know what it is that we need to do. If we don’t know what we need to do, well, what are we going to offer these girls, right?* [Non-objector, Mexico State, Mexico]

Many providers and other allied health personnel also spoke about the need to educate the general public, with special emphasis on outreach to women and girls. These responses fell into two categories: education for women and girls on their rights to legal abortion, and education for women and girls on pregnancy prevention. Notably, the belief that women and girls need more education and information on birth control was largely shared by objecting providers. This belief harkens to the perceived level of individual responsibility of patients seeking abortion care mentioned earlier, a responsibility largely placed on women and girls.

Less commonly, respondents made other suggestions, including: new laws and policies (especially in the case of Mexico, where state law and federal law and policy often conflict with one another), improved hospital-level policies, protocols, and procedures that guarantee abortion service provision at or near time of request, and dedicated spaces within hospitals for abortion care, staffed only by non-objecting providers and allied health personnel.

## Discussion

Our findings echo previous work that demonstrates that abortion-related CO is often misused to deny legal abortion services for reasons other than moral, religious, or ethical beliefs [4–8], and adds to the literature perspectives from non-objecting *and* objecting providers and other allied health personnel in Mexico and Bolivia. Overwhelmingly providers and other allied health personnel cited a lack of knowledge and understanding of abortion-related laws and policies and fear of legal problems from providing abortion services as the main reasons why providers object. Conversely, the main reason *to* provide services is to comply with the law, demonstrating that, for many, knowing and understanding the laws and policies and following them is reason enough to not object. This lack of familiarity, knowledge, and understanding about abortion laws is reinforced by the scarcity of legal abortion cases in many of these public hospitals; many providers spoke about the fact that they rarely encounter requests or cases of legal abortions in their facilities.

The belief that people who seek abortion care are irresponsible also permeated many of the interviews. Many providers and other allied health personnel view abortion as a last resort and/or the unfortunate result of the pregnant person not taking the necessary precautions by using contraception. This is closely tied to a lack of trust in people who seek abortion care, believing legal abortion to be a loophole through which irresponsible people who do not have a right to abortion under the law aim to access care.

Overall, providers and other allied health personnel identified a significant need for more education and training on abortion-related laws and policies in response to the lack of knowledge and fear of legal problems. The widespread fear of legal ramifications amplifies the need to provide physicians with additional resources and support to grow their confidence, knowledge, and clinical and non-clinical skills in providing legal abortion care. Advocates working to expand legal abortion access should recognize that lack of knowledge and understanding coupled with fear are the foremost reasons for refusing to provide legal abortion care in Mexico and Bolivia. These objectors are a key population who could provide abortion services if they are sensitized and equipped with the necessary resources to feel comfortable, confident, and protected.

Reasons for denying services as expressed by respondents in our study reinforce the reality that the existing practice of CO does not align with the intended exercise of CO as legally defined, nor with international human rights norms that uphold the primacy of the right to reproductive health care access over the individual exercise of CO. Further, our research reveals that CO is even appropriated by non-providers who do not have a legal right to object. This highlights the need for clearer and better disseminated regulation, both nationally and internationally, that clarifies the limits of CO and establishes processes for those who seek to claim CO. It also raises an important distinction in language: despite interviewees labelling individuals who refuse to participate in legal abortion services in certain situations as objectors, they are not objectors according to the legal definition. The language of CO is further complicated by the fact that the inclusion of the word *conscientious* implies that objectors are the ones with a conscience. However, many non-objecting providers link their provision of abortion services to their own conscience, which some researchers have encouraged calling *conscientious provision* [20, 21]. Advocates, researchers, and policymakers should understand this nuance. While respondents were not asked specifically about medication abortion (MA) as it relates to conscientious objection, it is important to acknowledge that MA has and continues to change the abortion landscape, both in terms of increased abortion access outside of health facilities and increased willingness of providers who may be more willing to administer MA than perform a surgical abortion procedure [22]. More research is needed to better understand the relationship between MA and the use of CO among health personnel in different settings, as well as implications for abortion service provision and CO regulation.

This study has limitations. For one, we cannot generalize findings to different countries or even to different states and departments within Mexico and Bolivia, respectively. However, our findings support research conducted in other settings, growing the evidence base about the understanding and use of CO around the globe. Secondly, all respondents work in public hospitals that have or had a working relationship with Ipas, and given receipt of Ipas training these hospitals may not be representative of other public hospitals with regard to abortion services. While not all respondents received Ipas training or technical assistance on abortion it is possible that the perspectives of health personnel in these public hospitals may differ from perspectives of personnel in facilities, public or otherwise, with no previous interaction with Ipas. That interviewer affiliation with Ipas was known to respondents may have contributed to social desirability bias; however, most respondents spoke candidly about their or their colleague’s refusal to provide or participate in abortion services. Finally, given that official registries of objecting providers do not exist in Mexico and Bolivia, recruitment of objecting and non-objecting providers was ultimately subject to error. Providers were recruited with the help of supervisors familiar with the objecting and non-objecting position of hospital staff, but in some cases, perceived objectors later self-identified as non-objectors and vice versa. Interviewers applied the appropriate interview guide as soon as the interviewee’s self-identified status was confirmed. Inclusion of objecting and non-objecting health personnel is a strength of this study.

## Conclusions

The main reasons for misuse of CO among our sample in Mexico and Bolivia were lack of knowledge of abortion law and fear of legal problems. This research is critical in contributing to the evidence base around CO globally and especially in Latin America, where misuse of CO represents a significant barrier to legal abortion access. It also identifies important ways to understand, reframe, and respond to the exercise of CO in Mexico and Bolivia, by recognizing that interventions aimed to increase understanding of abortion law and make providers feel protected in their provision of legal abortion would likely move the needle on misuse of CO in these settings.

## Data Availability

The transcripts of interviews with providers and allied health professionals analyzed in this study are not publicly available. The consent process with participants assured data would not be shared beyond the research team; in addition, many of the participants are abortion doctors working in sensitive settings. Data may be available from the corresponding author on reasonable request.

## Abbreviations

CO: Conscientious objection
IRB: Institutional Review Board
NOM 046: Norma Oficial Mexicana 046

## Footnotes

1 In Mexico City it is established that only physicians can perform abortions; in the rest of the country this is not established in the law but is an accepted practice.
